# Baseline Signal Reconstruction for Temperature Compensation in Lamb Wave-Based Damage Detection

**DOI:** 10.3390/s16081273

**Published:** 2016-08-11

**Authors:** Guoqiang Liu, Yingchun Xiao, Hua Zhang, Gexue Ren

**Affiliations:** 1 School of Aerospace Engineering, Tsinghua University, Beijing 100084, China; rengx@tsinghua.edu.cn; 2 Aircraft Strength Research Institute of China, Xi’an 710065, China; xiaoyc623@163.com (Y.X.); nantongzh1988@126.com (H.Z.)

**Keywords:** Hilbert transform, orthogonal matching pursuit, lamb waves, temperature compensation, damage detection

## Abstract

Temperature variations have significant effects on propagation of Lamb wave and therefore can severely limit the damage detection for Lamb wave. In order to mitigate the temperature effect, a temperature compensation method based on baseline signal reconstruction is developed for Lamb wave-based damage detection. The method is a reconstruction of a baseline signal at the temperature of current signal. In other words, it compensates the baseline signal to the temperature of current signal. The Hilbert transform is used to compensate the phase of baseline signal. The Orthogonal matching pursuit (OMP) is used to compensate the amplitude of baseline signal. Experiments were conducted on two composite panels to validate the effectiveness of the proposed method. Results show that the proposed method could effectively work for temperature intervals of at least 18 °C with the baseline signal temperature as the center, and can be applied to the actual damage detection.

## 1. Introduction

Recently, Lamb wave-based damage detection has received much attention from the research community because Lamb waves can travel over long distances and are sensitive to a range of types of damage including cracks, corrosion, and delaminations [1,2,3,4,5,6,7,8,9,10]. One commonly investigated Lamb wave-based damage detection paradigm uses a sparse array of permanently-affixed transducers. Typically, each transducer generates a Lamb wave while the others record the response, until a full measurement set of signals from every possible sensor pair is acquired. When Lamb wave-based damage detection is used to monitor the health condition of complex structures, the measured signals are often far too complex to be directly interpreted. The simple but effective way for damage detection is baseline subtraction approach, but environmental and operational conditions (EOC) can have a large impact on a measured wave. Variation in these conditions can cause errors in baseline subtraction. Among the EOC, temperature has been shown to be one of the dominant effects on the Lamb waves. It has been demonstrated that temperature can have an effect on the baseline subtraction signal as strong as damage [11].

In order to mitigate the temperature effect, many researchers have conducted theoretical and experimental investigations on the change in guided waves propagation caused by temperature fluctuations within the last decade [12,13,14,15,16,17]. Furthermore, several strategies for temperature compensation of the Lamb wave have also been developed in recent years. The optimal baseline selection (OBS) and baseline signal stretch (BSS) are the most commonly used methods. The OBS method selects the best matched waveform from a large baseline signals database collected from the structure at different temperatures [18,19]. The OBS method performs well but needs many baselines to obtain a sufficiently low post-subtraction noise level. This requirement is not always available in the engineering application. In contrast, the BSS method requires only one baseline to compensate the effect of temperature, which modifies a single baseline signal to match the current signal [20,21]. However, it is limited in the range of temperature change that can be accommodated. It has been shown that a combination of OBS and BSS provides an effective temperature compensation strategy and also allows reduction of the number of required baseline signals [22,23,24]. Besides the OBS and BSS, there are some temperature compensation methods which are not used for baseline subtraction but for weakening the temperature effect on the damage-sensitive features [25,26,27,28,29]. Recently, Roy et al. [30] proposed a physics-based approach for temperature compensation of piezoelectric sensor signals, which takes into account the influence of temperature on physical properties of base substrate, piezo-transducer, and adhesive interface. The drawback of the approach is that the algorithm needs training with prior data which are not always available. Wang et al. [31] use the combination of OBS and the adaptive filter to compensate the temperature variations. The simplistic representation of the signal and the choice of activation function are the main limitations of this approach. Fendzi et al. [32] present a data-driven temperature compensation approach which considers a representation of the piezo-sensor signal through its Hilbert transform that allows one to extract the amplitude factor and the phase shift in signals caused by temperature changes. The limitation of this method is that the compensation accuracy of the method depends on the length of the time window considered in the temperature compensation parameters estimation.

In addition, most temperature compensation methods for baseline subtraction need model training. However, it may not be possible to gather model training data for all possible combinations of structural and environmental changes. Furthermore, as far as we know, at present, the effectiveness of the temperature compensation methods are usually validated by the first few wave packets, and are rarely validated by the whole waveform. This validated temperature compensation method is not suitable for the damage detection situation that the damage is far from the sensing path.

This study presents a temperature compensation method based on baseline signal reconstruction. It compensates the baseline signal to the temperature of current signal. The Hilbert transform is used to compensate the phase of baseline signal. The Orthogonal matching pursuit (OMP) is used to compensate the amplitude of baseline signal. Experiments are conducted on two composite panels to validate the proposed method.

The remainder of this paper is organized as follows. Section 2 describes the proposed temperature compensation method. The temperature compensation validation without damage is introduced in Section 3. Then the damage detection validation is presented in Section 4. Finally, conclusions are presented in Section 5.

## 2. Temperature Compensation Method

In the baseline subtraction approach, the damage signal is the difference between the current signal and the baseline signal. So this temperature compensation method compensates the baseline signal to the temperature of current signal. It achieves the reconstruction of the baseline signal at the temperature of the current signal.

According to the literatures [25], the temperature-related shift of the Lamb wave signals can be observed as a change in instantaneous phase. According to the literature [31], the time of flight (TOF) of wave packets in Lamb wave signals have a good linear relationship versus temperature. So it supposes that the instantaneous phase difference of the two Lamb wave signals with different temperature is proportional to the temperature difference, with the limit range of temperature. This can be expressed by:

(1) $$arg\;s_{2}(t)-arg\;s_{0}(t)=\frac{T_{2}-T_{0}}{T_{1}-T_{0}}[arg\;s_{1}(t)-arg\;s_{0}(t)]$$

where $T_{0}$, $T_{1}$ and $T_{2}$ are the temperatures, $s_{0}(t)$, $s_{1}(t)$ and $s_{2}(t)$ are the Lamb wave signals at temperature $T_{0}$, $T_{1}$ and $T_{2}$ respectively, arg denotes the instantaneous phase of the signal.

According to the above-mentioned, the temperature-related shift of the Lamb wave signals can be compensated as follows. According to Equation (1), it needs two Lamb wave signals with different temperature before the compensation. Among the two Lamb wave signals, one is the baseline signal which is compensated to the temperature of the current signal, the other is the reference signal which together with the baseline signal is used to determine the relation between the instantaneous phase difference and the temperature difference. It assumes that $s_{b}(t)$ is the baseline signal at temperature $T_{b}$, $s_{r}(t)$ is the reference signal at temperature $T_{r}$, and $s_{c}(t)$ is the current signal at temperature $T_{c}$. Using the Hilbert transform, the instantaneous phase of the Lamb wave signal can be extracted. So compensating the instantaneous phase of the baseline signal $s_{b}(t)$ to the instantaneous phase of the current signal $s_{c}(t)$ can use the following formula:

(2) s˜b(t)=Re{(sb(t)+is^b(t))⋅eiϕ(t)}

where $\phi (t)=\frac{T_{c}-T_{b}}{T_{r}-T_{b}}[arg\;s_{r}(t)-arg\;s_{b}(t)]$, ${\hat{s}}_{b}(t)$ is the Hilbert transform of the baseline signal $s_{b}(t)$, Re{⋅} denotes the real part of the analytical signal, $i=\sqrt{-1}$ is the imaginary unit, ${\tilde{s}}_{b}(t)$ is the signal after instantaneous phase compensation of the baseline signal $s_{b}(t)$.

Then it needs to compensate the amplitude of ${\tilde{s}}_{b}(t)$ to the amplitude of the current signal $s_{c}(t)$. This problem is equivalent to represent the $s_{c}(t)$ by the ${\tilde{s}}_{b}(t)$. The problem can be expressed as:

(3) $$\min{\left\|s_{c}(t)-\alpha {\tilde{s}}_{b}(t)\right\|}_{2}^{2}$$

where α is the linear representation coefficient, ${\left\|\cdot \right\|}_{2}$ is the $\ell_{2}-$ norm of vector.

The problem of Equation (3) can be changed as:

(4) $$\min{\left\|s_{c}(t)-\beta {\bar{s}}_{b}(t)\right\|}_{2}^{2}$$

where β is the representation coefficient, ${\bar{s}}_{b}(t)$ is the unit $\ell_{2}-$ norm of ${\tilde{s}}_{b}(t)$. ${\bar{s}}_{b}(t)$ is calculated by:

(5) $${\bar{s}}_{b}(t)={\tilde{s}}_{b}(t)/{\left(\int_{-\infty }^{\infty }{\left({\tilde{s}}_{b}(t)\right)}^{2}dt\right)}^{1/2}$$

In order to solve the problem of Equation (4), the OMP [33] can be used. OMP is an iterative greedy algorithm, which gives a solution to the optimization problem:

(6) $$\underset{x}{\min}{\left\|s-Dx\right\|}_{2}^{2}\;subject\;to\;{\left\|x\right\|}_{0}\leq K$$

where s is given measurement vector, D is given dictionary, x is the sparse vector which is desirable to be recovered, the $\ell_{0}$ pseudo-norm ${\left\|x\right\|}_{0}$ is the number of nonzero components in x, and K is the assumed sparsity of x.

This study uses the OMP to calculate the representation coefficient β. ${\bar{s}}_{b}(t)$ is used as the only atom to form the dictionary. Using one iteration, the representation coefficient β can be expressed by:

(7) $$\beta ={\bar{s}}_{b}{\left(t\right)}^{†}s_{c}(t)={\bar{s}}_{b}{\left(t\right)}^{T}{\left({\bar{s}}_{b}(t){\bar{s}}_{b}{\left(t\right)}^{T}\right)}^{-1}s_{c}(t)=\langle {\bar{s}}_{b}(t),s_{c}(t)\rangle$$

where ${\bar{s}}_{b}{\left(t\right)}^{†}$ is the Moore-Penrose pseudoinverse of ${\bar{s}}_{b}(t)$, T denotes the vector transpose, −1 denotes the matrix inversion, 〈⋅,⋅〉 denotes the inner product operation.

Finally, the temperature compensation signal of the baseline signal $s_{b}(t)$ for the current signal $s_{c}(t)$ can be expressed by:

(8) $$s_{tc}(t)=\langle {\bar{s}}_{b}(t),s_{c}(t)\rangle {\bar{s}}_{b}(t)$$

where $s_{tc}(t)$ is the temperature compensation signal.

**Figure 1:**
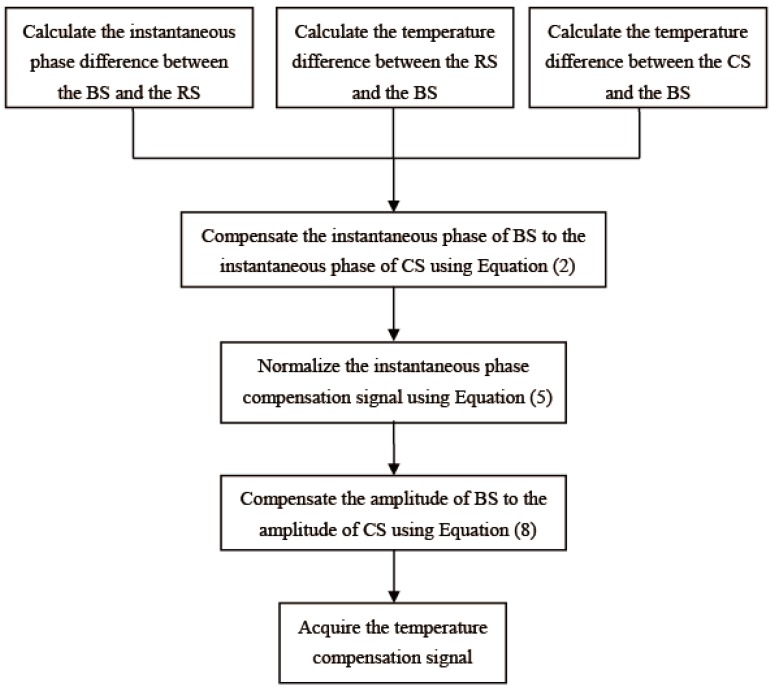
The flowchart of the proposed temperature compensation method. BS: baseline signal, RS: reference signal, CS: current signal.

Figure 1 shows the flowchart of the proposed temperature compensation method.

## 3. Temperature Compensation Validation without Damage

### 3.1. Experimental Setup and Procedure

**Figure 2:**
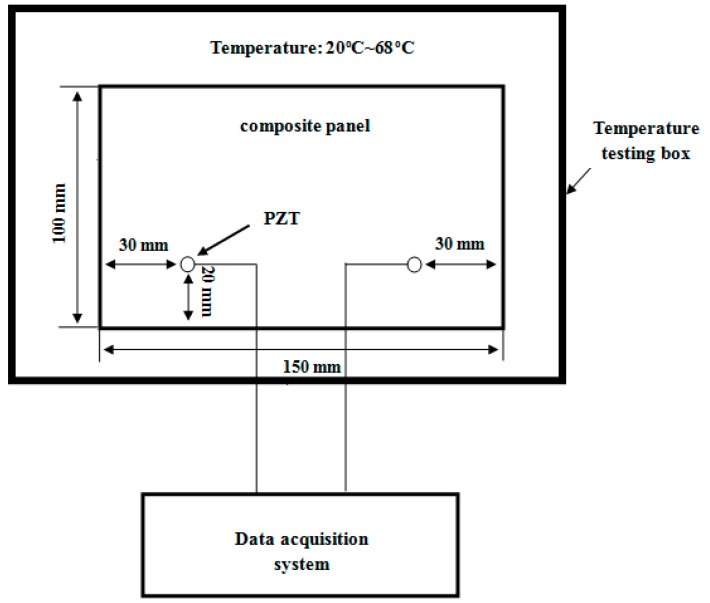
The schematic diagram of the PZT positions on the specimen used for temperature compensation validation without damage.

**Figure 3:**
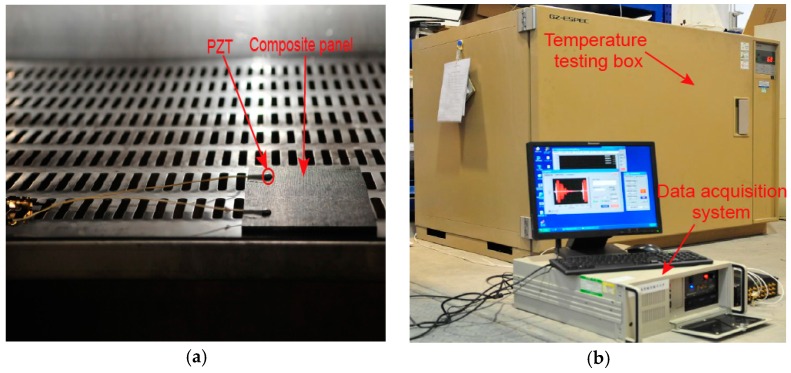
The experimental setup. (**a**) Specimen of composite panel; (**b**) Experiment test system.

The experiment was carried on a carbon fiber composite panel with the size of 150 mm × 100 mm × 3 mm. The material of composite panel is T700/BA9916. The stacking sequence is [45/0/−45/90/0/45/0/−45/0/45/90/−45]_S_. The thickness of each layer is 0.125 mm. Two Lead Zirconate Titanate (PZT) wafers were bonded on the specimen with the GLEIHOW302 adhesive. One was used as actuator, the other was used as sensor. The PZT wafers have a diameter of 8 mm and thickness of 0.45 mm. Their positions are shown in Figure 2. The specimen was put into a temperature testing box, where the highest temperature can reach 300 °C with 1 °C accuracy. The experimental setup is shown in Figure 3.

The experimental procedure is as follows: The temperature was gradually increased from 20 °C to 68 °C at the heating rate of 1 °C/min, and for every 3 °C increase, it was held for 20 min. From 20 °C, pitch-catch signal of the transducers was collected at every 3 °C increase. The excitation signal was a five-cycle tone burst with different center frequencies modulated by a Hanning window. Data were collected for central frequencies from 50 to 210 kHz with a step of 20 kHz. The sampling rate was 10 MHz. The collected signal length was 0.9 ms.

### 3.2. Results and Discussion

#### 3.2.1. Temperature Compensation Standard

To quantify the difference between two time-domain signals, $s_{x}(t)$ and $s_{y}(t)$, the error, Err, is introduced:

(9) $$Err=20\log(\frac{{\left\|\left(\left|s_{y}(t)-s_{x}(t)\right|\right)\right\|}_{\infty }}{{\left\|\left(\left|s_{x}(t)\right|\right)\right\|}_{\infty }})$$

where ${\left\|\cdot \right\|}_{\infty }$ is the $\ell_{\infty }-$ norm of vector.

In order to judge the temperature compensation results, $s_{x}(t)$ is the current signal and $s_{y}(t)$ is the baseline signal after the temperature compensation. According to common sense, the temperature compensation error should be bigger than the signal noise and smaller than the damage signal. So the lower limit of compensation standard is determined by comparing the two Lamb wave signals obtained in the health state and the damage state. The upper limit of the compensation standard is determined by the minimum ratio of the signal noise to the signal at different temperature. So we simulate the small damage of the composite panel by bonding a bolt with the diameter 5 mm on the panel surface at different position at room temperature. According to the statistical analysis of the experimental results, the typical Err value of residual signal caused by damage is −14.87 dB. The Err value of the minimum ratio of the signal noise to the signal at different temperature is −24.59 dB. Therefore, this study set the Err value as −22 dB for temperature compensation standard.

#### 3.2.2. Temperature Compensation Results

**Figure 4:**
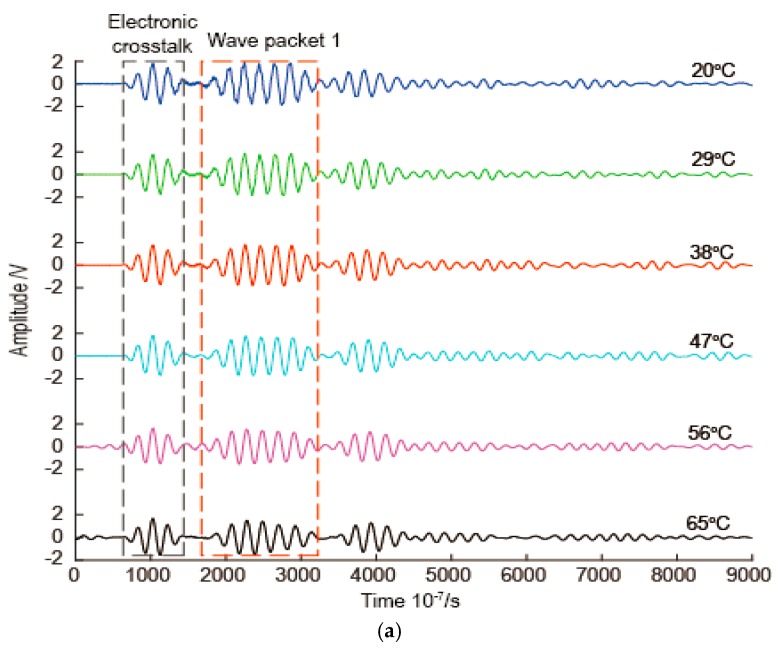

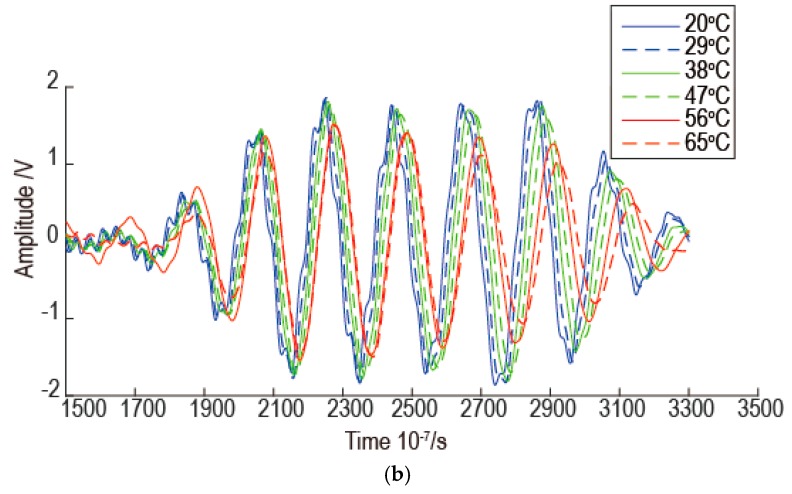
Signals with frequency 50 kHz at different temperatures. (**a**) A waterfall plot of the whole waveform; (**b**) Wave packet 1.

The change in temperature has effects on both signal amplitude and TOF for Lamb wave. For example, signals with frequency 50 kHz for the pitch-catch signal at different temperatures in this study are illustrated in Figure 4. Figure 4a is a waterfall plot of the whole waveform. As shown in Figure 4a, the electronic crosstalk is not affected by the temperature variation, but other parts of the signals are affected by the temperature variation. In order to more clearly observe the effects of temperature, the wave packet 1 of the signals are shown in Figure 4b. In Figure 4b, as the temperature increases, the signal amplitude decreases, and the TOF increases. The temperature compensation is used to decrease these changes caused by the temperature variations.

**Figure 5:**
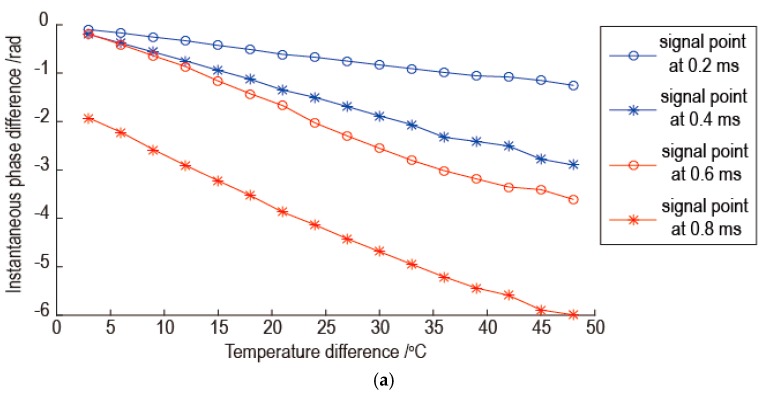

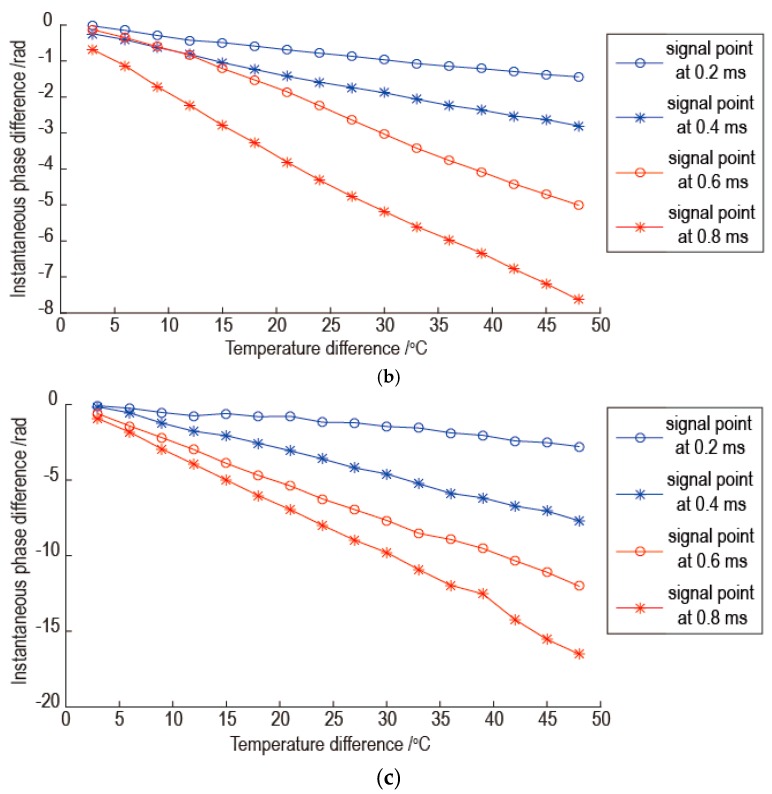
The relationships between the instantaneous phase difference and the temperature difference: (**a**) Signal with frequency 50 kHz; (**b**) Signal with frequency 130 kHz; (**c**) Signal with frequency 210 kHz.

In order to proof hypothesis of the proposed temperature compensation method, the relationship between the instantaneous phase difference and the temperature difference was studied. Using the instantaneous phase of the signals at 20 °C as benchmark, the instantaneous phase differences between signals at other temperatures and the signals at 20 °C were calculated. The calculated instantaneous phases of signals were unwrapped. The relationships between the instantaneous phase difference and the temperature difference for some signal points at three frequencies are shown in Figure 5. In Figure 5a, the signal points at 0.4 ms and 0.6 ms have relatively large deviation from linear relation between the instantaneous phase difference and the temperature difference. In Figure 5b, the signal point at 0.6 ms has a inflection point of linear relation between the instantaneous phase difference and the temperature difference. In Figure 5c, the signal point at 0.2 ms has many deviations from linear relation between the instantaneous phase difference and the temperature difference. From the overall view of Figure 5, although there is little deviation from the linear relation for some points, the instantaneous phase difference is proportional to the temperature difference. So the hypothesis of the proposed temperature compensation method is reasonable.

**Figure 6:**
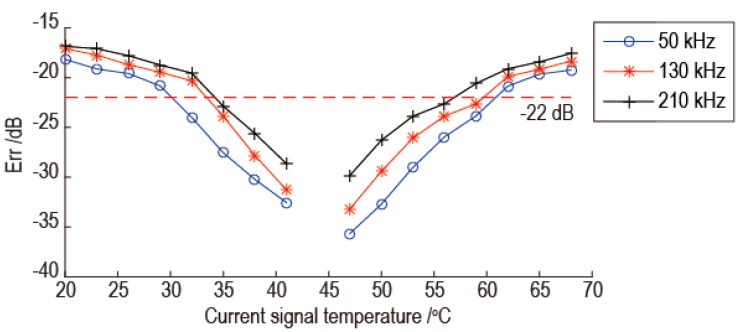
The temperature compensation results using 44 °C baseline signal and 47 °C reference signal.

**Figure 7:**
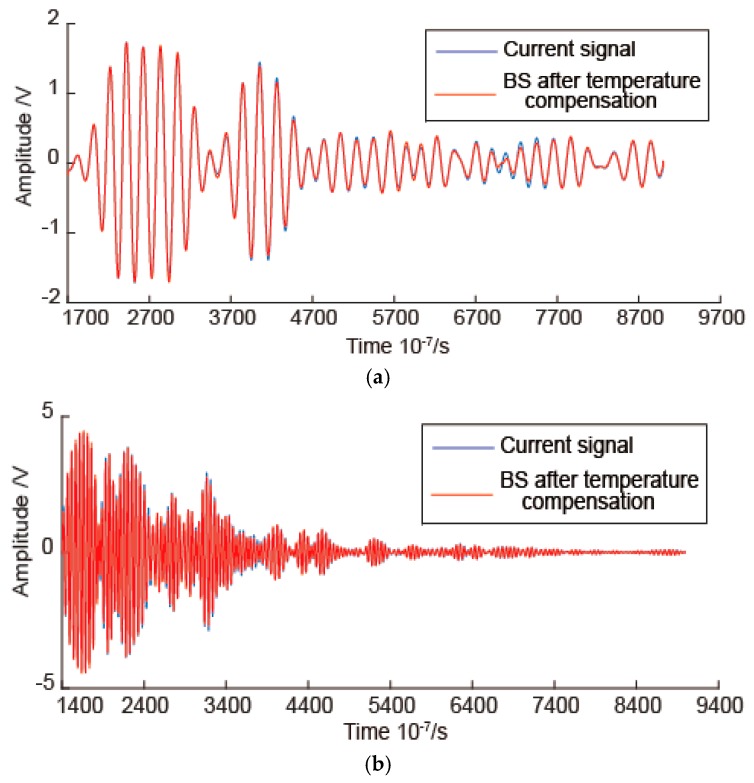
The comparison of 53 °C current signal and the temperature compensation signal of 44 °C baseline signal at two frequencies: (**a**) Signal with frequency 50 kHz; (**b**) Signal with frequency 190 kHz.

Before the temperature compensation, the experimental signals are processed by band pass filter, and the electronic crosstalk is removed. Then the experimental signals are processed by the proposed temperature compensation method using different baseline signals and reference signals. As an example, when the baseline signal temperature is 44 °C and the reference signal temperature is 47 °C, the temperature compensation results are shown in Figure 6. In Figure 6, the results for three frequencies are shown. As shown in Figure 6, the temperature compensation gets worse as the temperature difference between the current signal and the baseline signal increase. As the signal frequency increases, the temperature compensation gets worse and the temperature interval of the effective temperature compensation becomes smaller. The temperature interval of effective temperature compensation is at least 18 °C with the baseline signal temperature as the center. In order to see the result directly, for example, the temperature compensation results of 53 °C current signals with frequency 50 kHz and 190 kHz are shown in Figure 7. As shown in Figure 7a,b, the phase of baseline signal after temperature compensation is consistent with the phase of current signal. There are some slight amplitude differences between some parts of two signals.

**Figure 8:**
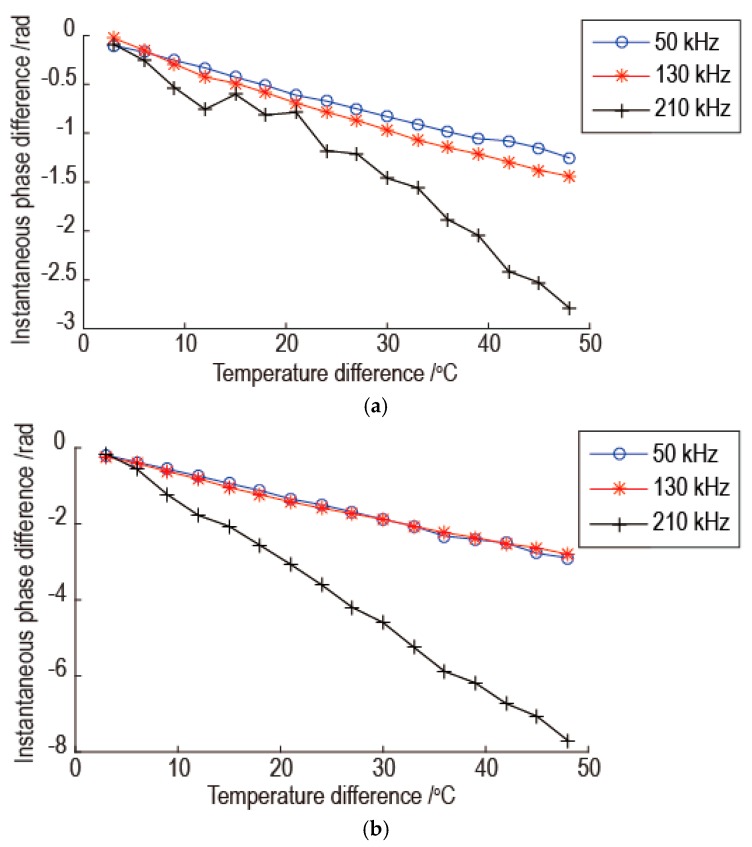
The comparison of the relationship between the instantaneous phase difference and the temperature difference for same signal point at different frequency: (**a**) Signal point at 0.2 ms; (**b**) Signal point at 0.4 ms.

The reasons for the results of Figure 6 are analyzed as follows. As shown in Figure 5, as the temperature difference between the current signal and the baseline signal increase, the proportional relation between the instantaneous phase difference and the temperature difference which is determined by the baseline signal and reference signal becomes worse. So the temperature compensation is worse as the temperature difference between the current signal and the baseline signal increase. As the signal frequency increases, there is more little oscillation for linear relation between the instantaneous phase difference and the temperature difference. This phenomenon can be revealed by comparing the relationship between the instantaneous phase difference and the temperature difference for same signal point at different frequency. Because the part of signal with large amplitude has a great influence on the error of temperature compensation, the signal points at 0.2 ms and 0.4 ms in Figure 5 are used for the comparison. The results are shown in Figure 8. As shown in Figure 8a, there are a few oscillations for linear relation between the instantaneous phase difference and the temperature difference for signals with frequencies of 50 kHz and 130 kHz, but there are many oscillations for signals with the frequency 210 kHz. The oscillation amplitude for signal with frequency 130 kHz is a little bigger than signal with frequency 50 kHz. The phenomenon in Figure 8b is the same with Figure 8a. So as the signal frequency increasing, the temperature compensation is worse and the temperature interval of the effective temperature compensation is reduced.

**Figure 9:**
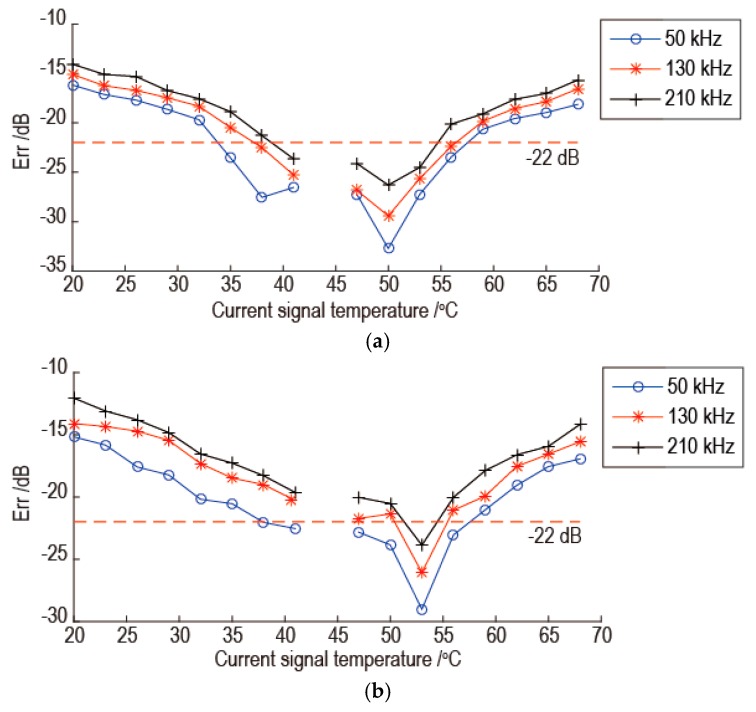
The temperature compensation results using 44 °C baseline signal and 50 °C, 53 °C reference signals: (**a**) The result using 50 °C reference signal; (**b**) The result using 53 °C reference signal.

In order to study the effect of temperature difference between the baseline signal and the reference signal on the temperature compensation, the baseline signal temperature is 44 °C and the reference signal temperatures are selected as 50 °C and 53 °C, respectively. The temperature compensation results are shown in Figure 9. By comparing the results of Figure 6 with Figure 9, it can be seen that as the temperature difference between the baseline signal and the reference signal increase, the Err of best temperature compensation at different frequency increases and the temperature interval of the effective temperature compensation at different frequency decreases. So it can be concluded that the temperature compensation is worse as the temperature difference between the baseline signal and the reference signal increase. This is because the error of proportional relation between the instantaneous phase difference and the temperature difference increase, as the temperature difference between the baseline signal and the reference signal increase. In Figure 9, when the current signal temperature is the same with the reference signal temperature, the temperature compensation is best. This is because the current signal is the same as the reference signal. So the instantaneous phase of the two signals is the same.

**Figure 10:**
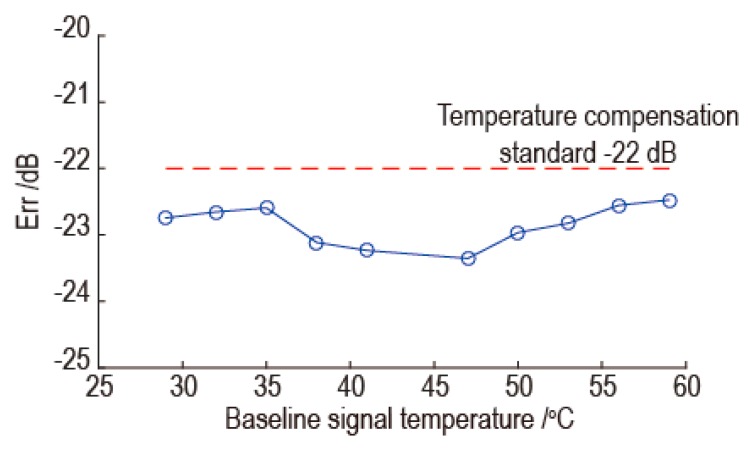
The worst temperature compensation results for each baseline signal for temperature interval 18 °C with the baseline signal temperature as the center.

According to the above results, the temperature compensation effectiveness of the proposed method is better when the temperature difference between the baseline signal and reference signal is small. In order to determine the temperature interval of effective temperature compensation, the temperature compensation was achieved by keeping the temperature difference between the reference signal and the baseline signal for 3 °C. Several baseline signals at different temperatures were used for temperature compensation, and the baseline signals are listed in Table 1. According to Figure 6, the temperature interval of effective temperature compensation was determined as 18 °C with the baseline signal temperature as the center. So the temperature interval of 18 °C with the baseline signal temperature as the center at different frequency was verified. The worst temperature compensation results for each baseline signal are shown in Figure 10. In Figure 10, the horizontal axis denotes each baseline signal at different temperatures, the vertical axis denotes the worst temperature compensation results for each baseline signal for temperature interval of 18 °C with the baseline signal temperature as the center at different frequency. As shown in Figure 10, the worst temperature compensation results for each baseline signal for temperature interval of 18 °C with the baseline signal temperature as the center, can satisfy the temperature compensation standard. So it can be concluded that the temperature interval of effective temperature compensation can be up to 18 °C.

## 4. Damage Detection Validation

**Figure 11:**
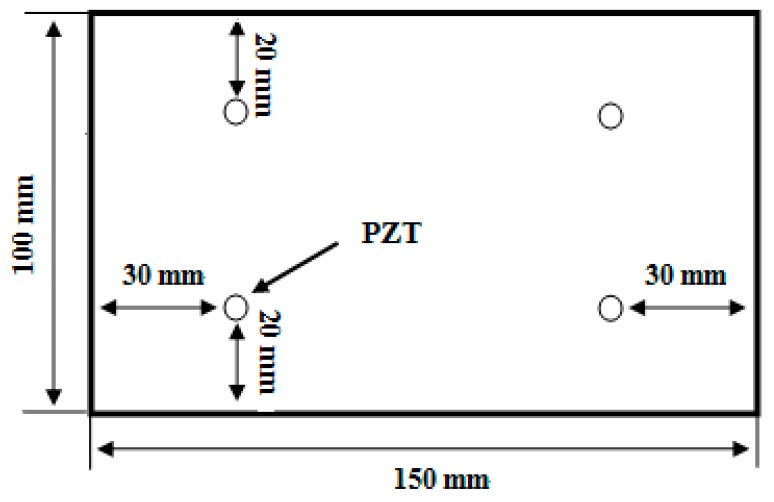
The schematic diagram of the PZT positions on the specimen used for damage detection validation.

The proposed temperature compensation method was also verified with damage detection on a carbon fiber composite panel which was the same with the Section 3. Four PZT wafers were bonded on the specimen. They worked in pitch-catch mode. Their positions are shown in Figure 11. The excitation central frequency was 70 kHz. The sampling rate was 10 MHz. The collected signal length was 0.9 ms. The experimental procedure is as follows:

(a) Baseline signals were collected at 18 °C without damage.
(b) Reference signals were collected at 20 °C without damage.
(c) Damage was produced by an impact on the specimen with a hammer.
(d) Environment temperature was increased to 26 °C.
(e) Current signals with damage were measured at 26 °C.

**Figure 12:**
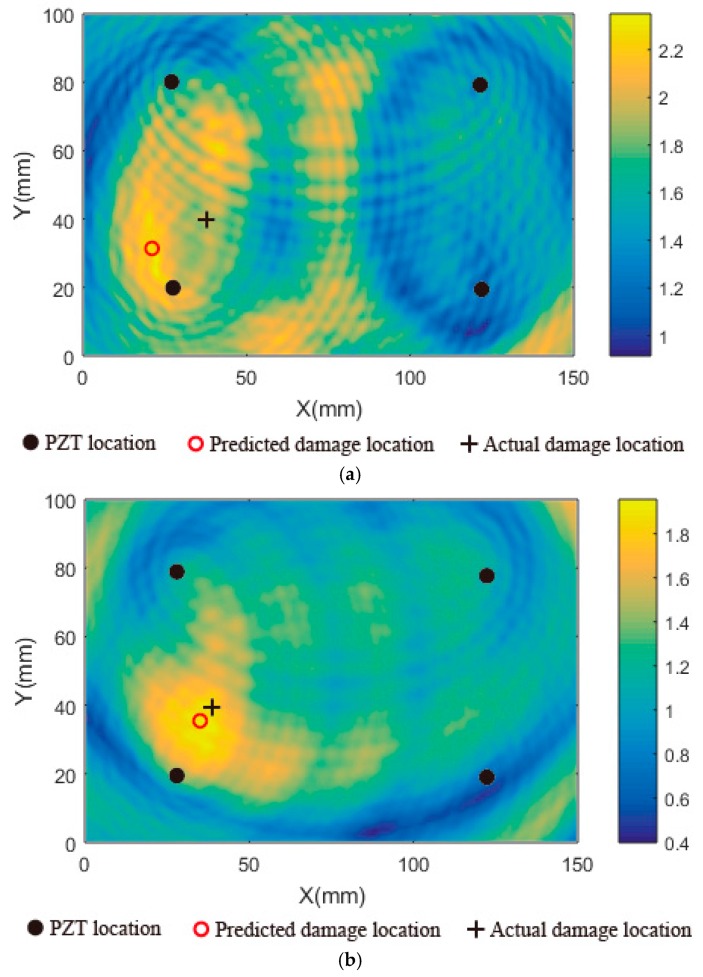
Damage imaging results: (**a**) Imaging result without temperature compensation; (**b**) Imaging result with temperature compensation.

The experimental signals were processed with the delay-and-sum damage imaging algorithm [34]. Under the 70 kHz excitation center frequency, A_0_ mode amplitude is dominant. So A_0_ mode was used for damage imaging. The damage detection results are shown in Figure 12. As shown in Figure 12a, the damage was imaged using the baseline signals at 18 °C and the signals obtained at 26 °C in the state with damage. According to the image, the damage location is not clear, and the damage imaging points are very scattered. The predicted damage location is far away from the actual damage location. When the baseline signals at 18 °C were compensated to the current signals at 26 °C using the proposed temperature compensation method, the damage imaging result is shown in Figure 12b. It is clear that the damage location can be determined according to the image result. The predicted damage location is almost consistent with the actual damage location. So it can be concluded that the proposed temperature compensation method can be applied to the actual damage detection.

## 5. Conclusions

In this paper, a temperature compensation method based on baseline signal reconstruction has been developed to enhance the robustness and effectiveness of Lamb wave-based damage detection. The method does not need model training. It only requires a baseline signal and a reference signal for temperature compensation. The effectiveness of the proposed method was validated by the experiments conducted on two composite panels. Results show that: (1) the instantaneous phase difference of the two Lamb wave signals with different temperature is proportional to the temperature difference, with the limit range of temperature; (2) the proposed method could effectively work for temperature intervals of at least 18 °C with the baseline signal temperature as the center; (3) the temperature compensation performance is degraded by the temperature difference between the baseline signal and the reference signal increase; (4) the damage signal extraction was effective after the temperature compensation; (5) the proposed method can improve the damage detection for temperature variation and can be applied to actual damage detection. In practical application, this method can be combined with OBS for damage detection.

## Figures and Tables

**Table 1:** The baseline signals and reference signals used for determining the temperature interval.

Baseline Signal (°C)	Reference Signal (°C)	Baseline Signal (°C)	Reference Signal (°C)
29	32	47	50
32	35	50	53
35	38	53	56
38	41	56	59
41	44	59	62
